# Proapoptotic effect and the mechanism of action of pingyangmycin on cavernous hemangiomas

**DOI:** 10.3892/etm.2013.1428

**Published:** 2013-11-26

**Authors:** YIDENG HUANG, PING LI, SIWEN XIA, YANG ZHUO, LONGJUN WU

**Affiliations:** 1 Department of Otorhinolaryngology, The 118th Hospital of Chinese PLA, Wenzhou, Zhejiang 325000, P.R. China; 2 Department of Otorhinolaryngology, General Hospital of Chengdu Military Region of PLA, Chengdu, Sichuan 610083, P.R. China

**Keywords:** pingyangmycin, proapoptotic, splenic cell

## Abstract

This study aimed to investigate the proapoptotic effects and the mechanism of action of pingyangmycin (PY) on cavernous hemangioma. The rat spleen was used as a model of cavernous hemangioma. PY was injected into the spleen and the pathological changes were observed at different time-points. Apoptosis was detected using terminal deoxynucleotidyl transferase dUTP nick end labeling (TUNEL) assay and transmission electron microscopy (TEM). The expression levels of the apoptosis-related protein, caspase-3, were determined using immunohistochemistry and image analysis. Rats injected with normal saline were the control group. Injection of normal saline did not damage rat spleens. On days 2 and 5 following PY injection, the spleens exhibited slight swelling. On days 8 and 14, atrophic changes were observed and the splenic sinus endothelial cells were damaged. At various time-points following PY injection, the apoptotic cells were observed by TEM. The TUNEL assay showed that apoptosis occurred widely among the splenic sinus endothelial cells and other splenic cells. The apoptotic rate and caspase-3 expression levels increased with prolonged PY exposure. PY induced apoptosis of splenic sinus endothelial cells through the caspase-3 activation pathway, and resulted in endothelial cell necrosis and fibroblast hyperplasia.

## Introduction

Hemangiomas mainly occur as benign tumors or arteriovenous malformation of the head and neck area (1). Removing these tumors is difficult and the surgical risks during resection are high. Intratumoral pingyangmycin (PY) injection has been demonstrated to be effective in treating tumors in clinical studies. The local reactions following PY injection were mild and the tumors spontaneously regressed (2–5). Previous studies have confirmed that PY induces apoptosis in malignant cells (6,7). Therefore, we speculated that PY may affect hemangioma endothelial cells through a similar mechanism, although no studies have confirmed this presumption. In the present study, hemangiomas were simulated using rat splenic tissues with extensive cavernous sinusoids. The possible mechanism of action and therapeutic targets of PY in hemangiomas were investigated. This study may provide a basis for further studies investigating potential treatments for hemangiomas. According to the research of Mulliken *et al*(1). Cavernous hemangiomas belongs to vascular malformations. The incidence in the head and the neck region is approximately 60% of the whole body (8). Pingyangmycin is a type of antitumor antibiotic which was first separated from the soil in Pingyang county of Zhejiang province, China in the last century, and its effects on the treatment of hemangioma are confirmed, but its mechanism of action remains unclear. It is suggested that pingyangmycin may effect the endothelial cells of the vessels. The major active component of pingyangmycin is bleomycin A5 (9). Pingyangmycin intralesion injection is a most widely used treatment for cavernous hemangioma (5,10).

## Material and methods

### Experimental animal model

A total of 54 Sprague-Dawley rats (male, n=27; female, n−27; weight 250–300 g) were provided by the Experimental Animal Center of the Second Military Medical University (Shanghai, China). The rats were randomly divided into nine groups (n=6 per group). The blank control group was not injected. The saline treatment group was injected with normal saline on days 2, 5, 8 and 14. Rats in the treatment group (PY group) were injected with 8 mg PY hydrochloride (Tianjin Taihe Pharmaceutical Co., Ltd., Hebei, China) in 5 ml normal saline on days 2, 5, 8 and 14. The animals were anesthetized with ketamine and diazepam (1:1) prior to injection. The skin on the left upper abdomen was disinfected, the abdominal wall was cut and the stomach was flipped over to show its dorsal side. The spleen was gently exposed and fixed in the abdominal cavity. Normal saline or PY solution (0.5 ml) was injected into the spleen along the longitudinal axis using a 1 ml syringe. The syringe was quickly retrieved following injection and slight pressure was applied to stop the bleeding. The wound was closed and the animals were placed in cages with 6 rats in each cage. The rats were maintained at 22–26°C in light and ventilated cages with water *ad libitum*. At 2, 5, 8 and 14 days after PY injection, rats were anesthetized, incisions were made along the original incisions and the spleen was removed by cutting the splenic vessels after ligation. Pathological changes of the spleen were observed through direct visualization. The head and tail of the spleen were removed, and the middle spleen was sectioned and fixed in 10% neutral formalin and glutaraldehyde. This study was carried out in strict accordance with the recommendations in the Guide for the Care and Use of Laboratory Animals of the National Institutes of Health (8th edition, 2012). The animal use protocol was reviewed and approved by the Institutional Animal Care and Use Committee (IACUC) of the 118th Hospital of Chinese PLA (Wenzhou, China).

### Tissue section preparation

The tissue specimens were fixed in 10% neutral formalin for 24 h, and subsequently dehydrated, cleared and embedded in paraffin. The tissue sections were stained with hematoxylin and eosin, and observed under a microscope (Leica ATC 2000; Beijing Guanpujia Technology Co., Ltd., Beijing, China).

Terminal deoxynucleotidyl transferase dUTP nick end labeling (TUNEL) was used to label the apoptotic cells. The TUNEL kit was provided by Roche Diagnostics (Shanghai) Co., Ltd. (Shanghai, China):

The paraffinized tissue sections (4 ml) were dewaxed with xylene, treated with 0.3% H_2_O_2_, digested with 20 μg/ml Proteinase K (Beyotime company, Shanghai, China) and labeled with TUNEL mixture for 30 min. Sections were washed with phosphate-buffered saline and the paraffinized sections were sealed with neutral resin (Shanghai Hualing Rehabilitation Equipment Factory, Shanghai, China).

### Immunohistochemical analysis of caspase-3 expression

Caspase-3 kit was provided by BD Biosciences Pharmingen (San Diego, CA, USA). The paraffinized sections (4 ml) were dewaxed using conventional xylene and subjected to antigen retrieval. The primary (Bcl-2 1:100, PcNA 1:200, F8 1:100, VEGF 1:60) and secondary antibodies (EnVision System) were added (Zhengzhou Biosail Technology and Trade Co., Ltd., Zhengzhou, China). The sections were stained with DAB (3,3′-dimethylbenzidine) and counterstained with hematoxylin. Tissue sections were differentiated using hydrochloric acid in ethanol, blued by washing with water and sealed with conventional resin.

### Microscopic image analysis

The paraffin sections subjected to TUNEL labeling and caspase-3 immunohistochemical staining were analyzed and photographed under a microscope (Axioplan 2 Imaging microscope and image analyzer; Carl Zeiss Microscopy GmbH, Göttingen, Germany). The measurement parameters were selected. The positive and strongly positive rates were calculated from three replicates in each group. Three visual fields were examined for each section.

### Transmission electron microscopy (TEM)

Specimens were fixed with glutaraldehyde solution for 2 h, refixed with 1% osmium tetroxide, dehydrated with ethanol and acetone, and embedded in Epon 812 epoxy resin (Hede Biotechnology Go., Ltd., Beijing, China). A 50–70-nm thin section was obtained and subsequently dyed with uranium lead staining. Apoptotic cells were observed and photographed under an XP-201 transmission electron microscope (Chongqing Mic Photoelectric Instrument Co., Ltd., Chongqing, China).

### Statistical analysis

The mean of multiple samples were analyzed using SPSS software, version 11.0 (SPSS Inc., Chicago, IL, USA). P<0.05 was considered to indicate a statistically significant result.

## Results

### Morphological observations

The spleens of the rats in the control group were dark red, with a smooth surface and evident swelling. The spleens of the rats in the saline group did not significantly differ from those of the control group. The splenic tissues from the PY day 2 group were dark red and showed slight swelling. The splenic tissues from the PY day 5 group were dark red and the surface was slightly concave with mild swelling. The splenic tissues from the PY day 8 group exhibited atrophy, with jagged edges, a white scar on the surface and adhesion in the surrounding tissues. The splenic tissue from the PY day 14 group showed atrophy, with a white scarring and depressions on the surface, and abundant adhesion in the surrounding tissues.

### Light microscope observations

The appearance of the splenic tissues in the control and saline groups were the essentially the same with no significant histological differences. The splenic sinuses in the PY day 2 group were dilated and congested, with several degenerated sinusoidal endothelial cells and splenic cord fiber cells. The sinusoidal endothelial cells were swollen with eosinophilic changes of nuclear condensation and fragmentation. The structure of the splenic cord was slightly blurred with infiltrating inflammatory cells and histiocytic hyperplasia. The splenic tissue from the PY day 5 group was congested with significant expansion. A number of sinusoidal endothelial cells and splenic cord fiber cells were degenerated and the sinusoidal endothelial cells were swollen with eosinophilic changes. Numerous cells showed nuclear condensation, fragmentation and disintegration, and the structure of the splenic cord was indistinct. Many inflammatory cells showed infiltration and histiocytosis. The spleen sinus endothelial cell structure was not observed in the PY day 8 group, but nuclear dissolution, fibrin exudation and partial shrinkage of splenic bodies were observed. The edges were congested with bleeding. The splenic corpuscles were atrophied in the PY day 14 group, with dead endothelial cells and fibrous tissue proliferation. The splenic capsule was thickened and interstitial hemosiderin deposition was observed.

### TUNEL assay and caspase-3 immunohistochemical analysis

**Figure 1:**
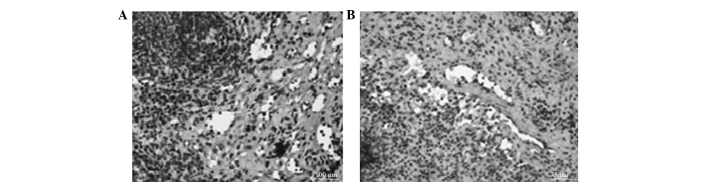
Apoptosis and caspase-3 expression in the spleen 5 days following PY injection. (A) TUNEL assay showed positive staining in the spleen sinusoid endothelial cells and other cells. Image analysis indicated a positive rate of 31.44% (strongly positive rate was 18.36%). (B) Immunohistochemical analysis showed caspase-3 expression in the spleen sinusoid endothelial cells. The positive rate was 60.55% (strongly positive rate was 27.24%). PY, pingyangmycin; TUNEL, terminal deoxynucleotidyl transferase dUTP nick end labeling.

TUNEL labeling identified positive staining in the cytoplasm (Fig. 1A). The control and saline groups showed a small degree of positive staining. As the PY exposure was prolonged, the splenic sinus endothelial cell apoptosis increased. Caspase-3 immunohistochemistry indicated positive staining in the cytoplasm (Fig. 1B). Caspase-3 expression was also observed in the sinusoidal endothelial cells of the control and saline groups. Following PY treatment, increased staining was observed in the splenic stromal cells, lymphocytes and vascular endothelial cells. The control group was selected for comparison with the day 2 and day 5 saline groups, as well as the day 2 and day 5 PY groups as the sinusoids were destroyed on days 8 and 14. The results of the microscopic image analysis showed no significant differences between the day 2 and day 5 saline groups and the control group, whereas the PY day 2 and day 5 groups showed significantly higher staining than was present in the control group (P<0.01; Table I).

### TEM observations

**Figure 2:**
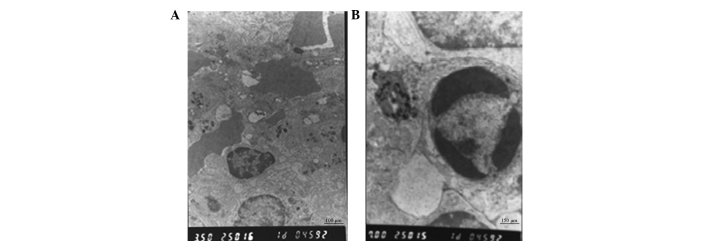
TEM image analysis of splenic tissues 5 days following PY injection. (A) Red blood cells and blood platelet coagulation were observed and thrombus formed. (B) Apoptotic cells were observed in the spleen tissue. TEM, transmission electron microscopy; PY, pingyangmycin.

The structure of the splenic sinusoids was normal in the control and saline groups at the various time-points. In the PY day 2 group, the splenic sinus was dilated and congested, with several degenerated sinusoidal endothelial cells and splenic cord fiber cells. The sinusoidal endothelial cells were swollen with eosinophilic changes, and the nucleus showed condensation and fragmentation. The structure of the splenic cord was indistinct and a few infiltrating inflammatory cells with histiocytic hyperplasia were observed. The splenic tissues from the PY day 5 group were significantly congested, and the splenic sinuses were expanded and congested, with numerous degenerated sinusoidal endothelial cells and splenic cord fiber cells. The sinusoidal endothelial cells were swollen with eosinophilic changes, and nuclear condensation, disintegration and fragmentation were observed (Fig. 2). The structure of the splenic cord was indistinct and a few infiltrating inflammatory cells with histiocytic hyperplasia were observed. The splenic sinusoids and cell structure of the PY day 8 group were not present, demonstrating nuclear dissolution. Several nuclei were observed along the edge of the cells, with fibrin exudates and shrinkage of certain splenic bodies. Additionally, fibrous tissues indicated proliferation with congestion and hemorrhage along the edge. The splenic bodies in the PY day 14 group showed atrophy, the splenic sinuses were collapsed, the endothelial cells and interstitial cells were necrotic, with fibrous proliferation, the spleen capsule was thick and interstitial hemosiderin deposition were observed.

## Discussion

No animal model of cavernous hemangioma is available for study. In previous studies, rats have been injected with tumor endothelial cells to induce the formation of vascular tumors, but the resulting vascular tumors significantly differed from hemangiomas (11,12). The spleen exhibits extensive sinusoids, which are large and irregular with mutual connections and filled with blood. The sinus walls consist of rod-shaped endothelial cells arranged along the longitudinal axis of the sinusoids and lined with venous sinusoids of the cavernous hemangioma. These features are similar to those of normal venous endothelial structures. Thus, the spleen was chosen as a model for studying the mechanism of action of PY for treating cavernous hemangioma. In the present study, morphological and TEM ultrastructural observations indicated that the splenic sinus endothelial cells and other cells of the spleen were impaired under PY treatment and the splenic sinuses were gradually destroyed. The complete splenic sinus structure was unclear under light microscopy and electron microscopy following PY injection on days 8 and 14. Among the methods for analyzing apoptotic cells, observation of morphological changes under an electron microscope was the most credible (13). Apoptotic cells were observed at varied PY treatment times under an electron microscope; however, apoptotic cells were not identified in the control group. A certain degree of necrosis was observed by light and electron microscopy. Marginal fibroblast proliferation was observed 8 and 14 days after PY injection. The morphological changes may be associated with the splenic capsule thickening and fibrosis proliferation observed by light microscopy.

TUNEL assay is the most sensitive, rapid and specific method for detecting apoptosis *in situ*(14,15). TUNEL assays are widely used for detecting apoptosis due to the specificity of the results and the accurate expression of morphological characteristics, localization and distribution of apoptotic cells. Furthermore, small concentrations of apoptotic cells may be detected (16). In the present study, the analysis of splenic sinus vascular endothelial cell apoptosis using a TUNEL assay showed that apoptosis increased in the spleen sinusoidal endothelial cells, stromal cells and lymphocytes on days 2 and 5 following PY treatment. Image analysis indicated that the apoptotic rate was significantly higher than that in the control and saline groups. These results are consistent with the gradual destruction of the spleen structure observed by light and electron microscopy and demonstrated that apoptosis is the main cause of structural damage in splenic tissues treated with PY.

Caspase-3 is a member of the caspase family and an important initiator and executor of apoptosis (17). Caspase-3 triggers the characteristic nuclear changes of apoptosis, such as chromatin condensation and DNA cleavage (18). The apoptosis of hemangioma endothelial cell has been identified to be caspase-dependent in a clinical study (19). In the present study, quantitative analysis showed that caspase-3 expression was positive in the control group, which may be associated with the presence of a few apoptotic cells in the spleen (20). However, caspase-3 expression gradually increased with the extension of PY action time, which corresponded with the increased rate of PY-induced apoptosis.

In conclusion, PY was observed to induce the apoptosis of endothelial cells in the splenic sinuses, which was accompanied by a certain degree of necrosis and fibroblast proliferation, ultimately resulting in the destruction of splenic sinuses, splenic atrophy and scarring. Apoptosis induced by PY treatment is associated with increased caspase-3 activity. Considering cell necrosis usually does not exhibit caspase-3 activation characteristics (21), the results indicated that PY induced apoptosis through the caspase-3 activation pathway.

The clinical treatment of cavernous hemangioma with PY requires repeated injections. Therefore, the aforementioned effects may be more intense in the clinic.

The induction of apoptosis may become the primary method for treating tumors (22,23). As caspase activation induces apoptosis (24,25), the caspase-3-dependent induction of apoptosis in spleen tissues by PY may provide a novel method for treating hemangiomas. Caspase-3 was specifically activated to trigger apoptosis and the apoptotic efficiency of tumor vascular endothelial cells was further improved (26). Hemangioma regression was performed to avoid the repeated use of chemotherapy agents that may result in adverse reactions.

## Figures and Tables

**Table I:** Changes in the rate of apoptosis and caspase-3 expression levels following PY injection (%).

	Apoptosis		Caspase-3	
		
Group	Positive rate	Strongly positive rate	Positive rate	Strongly positive rate
Control	13.71±2.97	5.89±1.26	24.52±1.43	4.34±1.92
Saline (day 2)	15.35±1.14	6.57±0.79	23.70±1.88	4.78±0.78
Saline (day 5)	14.08±2.96	5.75±1.04	23.61±2.97	4.92±1.45
PY (day 2)	27.91±3.18^a^–^c^	16.16±2.41^a^–^c^	59.76±3.37^a^–^c^	32.03±0.31^a^–^c^
PY (day 5)	32.50±4.07^a^–^c^	19.00±3.49^a^–^c^	63.23±3.27^a^–^c^	30.91±3.23^a^–^c^

Data are the mean ±standard deviation. n=3 per group.

a P<0.01 vs. the control group;

b P<0.01 vs. the saline day 2 group;

c P<0.01 vs. the saline day 5 group.

PY, pingyangmycin.
